# Lianhua Qingwen Capsules Reduced the Rate of Severity in Patients with COVID-19: A System Review and Meta-Analysis of Randomized Controlled Trials

**DOI:** 10.1155/2022/9617429

**Published:** 2022-02-02

**Authors:** Chengqian Shi, Mizhi Wu, Kepeng Yang, Xinchang Wang

**Affiliations:** ^1^Department of Rheumatology, The Second Affiliated Hospital, Zhejiang Chinese Medical University, Hangzhou 310005, China; ^2^Department of General Surgery, Sir Run-Run Shaw Hospital, Zhejiang University School of Medicine, Hangzhou 310016, China

## Abstract

**Background:**

The rate of severity is a critical factor affecting the prognosis and mortality in coronavirus disease 2019 (COVID-19). Lianhua Qingwen capsules or granules (LQ) have been a promising Chinese patent medicine in treating infectious diseases and recommended for treating COVID-19. This meta-analysis aims to demonstrate the association between LQ treatment and the rate of severity in patients with mild or moderate COVID-19.

**Methods:**

7 electronic databases were systematically searched from the inception dates to March 27, 2021, using the search terms to identify randomized controlled trials (RCTs). Two reviewers independently identified studies, extracted the data, and assessed study quality. All analyses were conducted on RevMan 5.3 software.

**Results:**

A total of 5 RCTs involving 830 patients with mild or moderate COVID-19 were identified according to the inclusion and exclusion criteria. The quality of included studies is moderate. Compared with conventional therapy, there was a significant association of LQ treatment with a higher clinical efficacy (RR = 1.24, 95% CI (1.13, 1.36), *P* < 0.00001), rate of CT improvement (RR = 1.22, 95% CI (1.10, 1.34), *P*=0.0001), and a lower rate of conversion to severe cases (RR = 0.47, 95%CI (0.31, 0.71), *P*=0.0003).

**Conclusion:**

LQ combined with conventional therapy had great effects in reducing the rate of severity, and these findings supported the routine treatment of LQ in patients with mild or moderate COVID-19.

## 1. Introduction

The coronavirus disease 2019 (COVID-19) pandemic, caused by severe acute respiratory syndrome coronavirus 2 (SARS-CoV-2), has led to a global health crisis [1]. The most common symptoms in patients with mild COVID-19 are fever, cough, fatigue, and in severe cases, dyspnea, bilateral lung infiltration, and hemodynamic instability [1, 2]. The rate of severity is a critical factor affecting the prognosis and mortality in patients with mild or moderate COVID-19. As of April 2021, there have been more than 130 million infections and 2.5 million deaths around the world. In the absence of specific antiviral drugs [3], vaccines are considered an essential countermeasure directed against SARS-CoV-2 that causes COVID-19. However, it is so far to clarify a safe and immunologically efficient strategy of COVID-19 vaccine [4, 5].

There are more than 300 ongoing clinical trials to identify effective therapies for prevention and treatment, but only limited essential drugs and restricted access to health facilities in treating COVID-19 at the present [3]. Differently, the application of traditional Chinese medicine (TCM), including Chinese herbal decoctions, Chinese patent medicines (CPM), acupuncture, and other TCM treatments, is inspired in the treatment of patients with COVID-19 in China, and the total effective rate is over 90% [6, 7]. Lianhua Qingwen capsules or granules (LQ), one kind of CPM, play an important role in the treatment of serious epidemic diseases, particularly influenza [8]. It is a manufactured product of TCM (Lianqiao, Jinyinhua, Zhimahuang, Chaoxingren, Shigao, Banlangen, Guanzhong, Yuxingcao, Guanghuoxiang, Dahuang, Hongjingtian, Bohenao, and Gancao) that could significantly inhibit proliferation of virus and exert anti-inflammatory activity [9]. Based on the previous experience with TCM theory and the Diagnosis and Treatment Guideline for COVID-19 (Trial 8th Edition), LQ has been widely applied to the treatment of COVID-19 with remarkable therapeutic effects [9, 10]. However, meta-analyses published have not reached a convincing conclusion that the LQ treatment is associated with the rate of severity in patients with COVID-19.

Patients with mild or moderate COVID-19 lacking medical treatment have a higher risk of conversion to severe cases compared with patients receiving medical treatment [11, 12]. Therefore, the rate of severity may differ between LQ with conventional therapy and conventional therapy. We performed a systematic review and meta-analysis to investigate and compare LQ combined with conventional therapy and conventional therapy for the rate of severity in patients with mild or moderate COVID-19.

## 2. Methods

### 2.1. Searches

Studies were identified from 7 electronic databases (4 in English and 3 in Chinese): PubMed, Embase, Web of Science (WOS), Cochrane Library, China National Knowledge Infrastructure (CNKI), China Biology Medicine (CBM), and Wanfang. Search terms included “COVID-19,” “SARS-CoV-2,” “2019-nCoV,” “Novel coronavirus pneumonia,” “lianhua qingwen,” and “lianhuaqingwen.” The searching time was from the inception dates to 2021-03-27. The protocol had been registered on PROSPERO (no. CRD42021245523).

### 2.2. Study Inclusion and Exclusion Criteria

Studies were selected based on the following criteria: (1) randomized controlled trials (RCTs); (2) participants were diagnosed as mild or moderate COVID-19; (3) LQ combined with conventional therapy treatment (LQ group) or conventional therapy only (control group); (4) relevant efficacy index (clinical efficacy, improvement of chest computer tomography (CT), rate of conversion to severe cases, rate of mortality, and adverse reactions). The following types of studies were excluded: (1) observational studies; (2) the protocol of meta-analysis; (3) trials were given to more than two Chinese patent medicines in one group at the same time; (4) trials were given to more than two Chinese patent medicines in one group at the same time; (5) animal and other nonclinical trials.

### 2.3. The Criteria of Severe Cases

The criteria of severe cases in included studies should meet one of the following criteria: (1) progressive exacerbation of hypoxemia or respiratory distress; (2) deterioration of tissue oxygenation index or progressive increase of lactic acid; (3) progressive decrease of peripheral blood lymphocyte count or increase of peripheral blood inflammatory markers; (4) significant abnormal changes in coagulation function related indicators; (5) significant progression of lung disease is indicated by chest radiological imaging.

### 2.4. Data Extraction and Quality Assessment

Two researchers (C. Q. S. and M. Z. W.) independently extracted the following information from the included studies: lead author, year of publication, country of origin, sample size, age, gender, disease severity, interventions, and treatment duration. Two researchers (C. Q. S. and K. P. Y.) independently assessed the quality of the included studies by the Cochrane Collaboration risk of bias assessment tool. Disagreements were resolved by the third reviewer (X. C. W).

### 2.5. Statistics Analysis

Dichotomous variables were calculated by relative risk (RR) with 95% confidence interval (CI), and continuous variables were calculated by weighted mean difference (WMD) with 95% CI. The *I*^2^ statistic was calculated to indicate heterogeneity: *I*^2^>50% indicated significant heterogeneity between the included studies, and a random-effects model was used to display the data if the heterogeneity failed to be identified by subgroup analysis or sensitivity analysis. Potential publication bias risk was assessed with funnel plots. Data were analyzed with Review Manager 5.4.1, and a *p* value <0.05 was considered significant.

## 3. Result

### 3.1. Search Results

**Figure 1:**
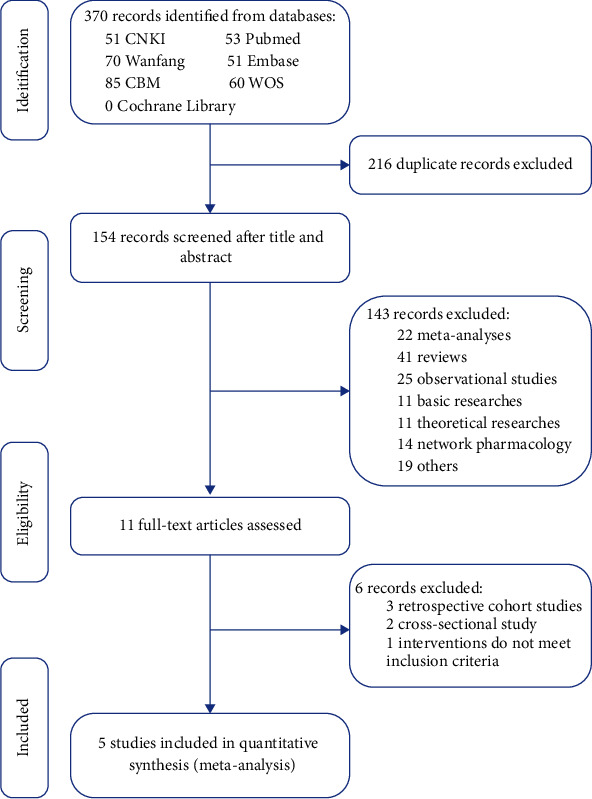
Literature search and screening process.

From the searches for 7 electronic databases, 370 eligible records were identified. Duplicating, screening the title and abstract, and assessing the full-text according to the inclusion and exclusion criteria, 365 records were excluded for various criteria. Finally, a total of 5 studies [13–17] were included in this meta-analysis (Figure 1).

### 3.2. Characteristics of Included Studies

All included studies were RCTs and conducted in China. Among the included studies, 3 were published in Chinese [13–15] and 2 were in English [16, 17].

A total of 5 RCTs [13–17] involving 830 patients (464 males and 366 females) were included in these studies, and 3 patients dropped out (2 in the LQ group and 1 in the control group) [15] in the end. 412 (49.6%) patients involved in LQ treatment in combination with conventional therapy (such as interferon-*α*, lopinavir/ritonavir, arbidol, and other antivirals). The formulation of LQ included capsules (0.35 g/capsule) and granules (6 g/bag). The duration of treatment varied from 7 to 15 days (Table 1).

### 3.3. Study Quality

**Figure 2:**
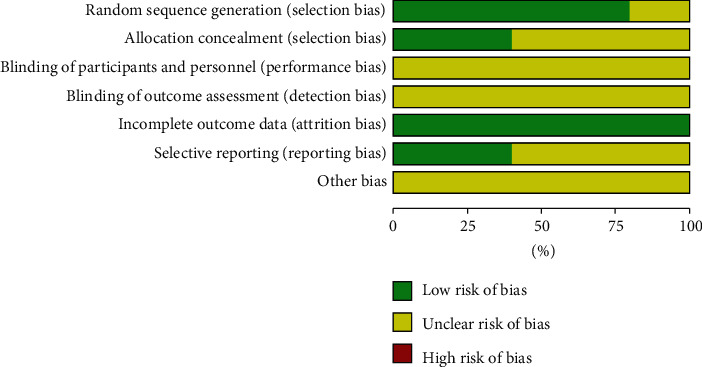
The quality of included studies.

The quality of included studies is shown in Figure 2. Four studies [14–17] described the random sequence generation, and two studies [16, 17] described the methods used for allocation concealment. None of the study described the blinding of participants, personnel, and outcome assessment. Five studies [13–17] adequately addressed incomplete outcome data, and two studies [16, 17] registered the protocol and reported the registration information. Overall, the quality of included studies is moderate.

### 3.4. Clinical Efficacy

**Figure 3:**
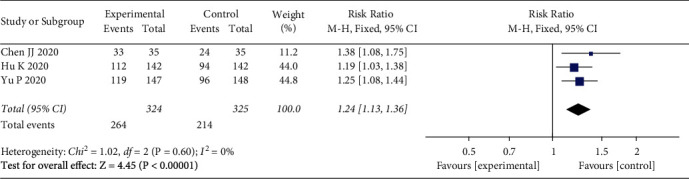
Clinical efficacy.

3 studies [13, 14, 16] involving 649 patients with COVID-19 compared the clinical efficacy between the LQ group and control group. There were low levels of heterogeneity (*I*^2^ = 0%, *P*=0.06) between studies for clinical efficacy, and a fixed effects model was used for the analyses. The results showed that the clinical efficacy in the LQ group was higher than in the control group (RR = 1.24, 95% CI (1.13, 1.36), *P* < 0.00001) (Figure 3).

### 3.5. Rate of CT Improvement

**Figure 4:**
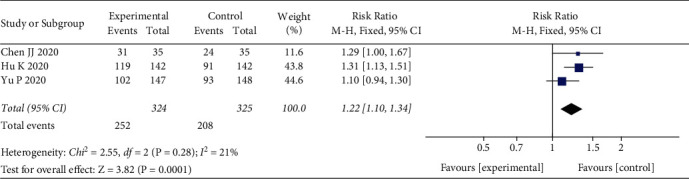
Rate of CT improvement.

3 studies [13, 14, 16] involving 649 patients with COVID-19 compared the rate of CT improvement between the LQ group and control group. The heterogeneity between studies was not significant (*I*^2^ = 21%, *P*=0.28), and the results were pooled using a fixed effects model. The outcomes indicated that the rate of CT improvement in the LQ group was better than in the control group (RR = 1.22, 95% CI (1.10, 1.34), *P*=0.0001) (Figure 4).

### 3.6. Rate of Conversion to Severe Cases

**Figure 5:**
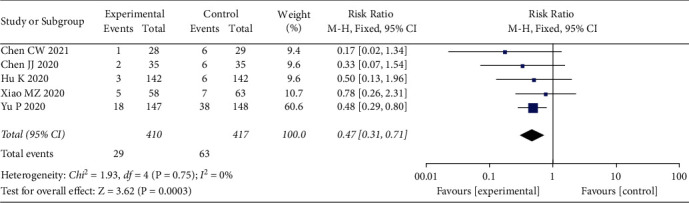
Rate of conversion to severe cases.

5 studies [13–17] involving 827 patients with COVID-19 compared the rate of conversion to severe cases between the LQ group and control group. Heterogeneity between included studies was considered not significant for the analyses of the rate of conversion to severe cases (*I*^2^ = 0%, *P*=0.75), and a fixed effects model was used for pooling the results. Meta-analysis strongly indicated that the rate of conversion to severe cases in the LQ group was lower than in the control group (RR = 0.47, 95%CI (0.31, 0.71), *P*=0.0003) (Figure 5).

### 3.7. Publication Bias

**Figure 6:**
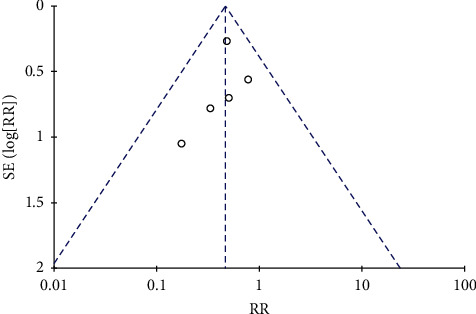
Funnel plots.

Funnel plots for the meta-analysis of the rate of conversion to severe cases are shown in Figure 6. Five studies were enrolled into this analysis, and the plots indicated mild publication bias among the included studies.

## 4. Discussion

On 11 February 2020, the Chinese Center for Disease Control and Prevention reported 44,672 confirmed cases (257 drop-out cases), including 36,160 (80.9%) mild cases, 6,168 (13.8%) severe cases, and 2,087 (4.7%) critical cases with 1,023 (2.3%) deaths [18]. The rate of severity in patients with COVID-19 is approximately 20%, and the severity is difficult to decrease if medical resources are insufficient in mild or moderate cases. Therefore, it is essential for COVID-19 pandemic control and elimination to select an effective and reproducible therapy to decrease the rate of conversion of severe cases.

Currently, there is no high-level evidence from RCTs that any specific drugs target SARS-CoV-2 in patients with COVID-19 [1, 3]. Actually, TCM is applied in clinics early in China and has different effects on the distinct stages of patients with COVID-19 [6, 9]. LQ, patented in 2003 in China and approved for Phase II clinical trial by US FDA in 2005, has worked well in treating infection disease and playing an important role in treating COVID-19 [7, 10]. Yang et al. [19] reported that LQ displayed the effectiveness of antiviral and inhibited the mRNA expression of inflammatory cytokines (RANTES, IL-6, IL-8, IP-10, TNF-*α*, MCP-1, MIP-1*β*, and IFN-*λ*) against influenza B infection in the mouse model. Ding et al. [20] reported that LQ exerted broad-spectrum effects on various influenza viruses by inhibiting viral propagation and impacting immune function. Furthermore, Duan et al. [8] reported that LQ, compared with oseltamivir, achieved a similar therapeutic effectiveness reduction of the duration of illness and duration of viral shedding in patients with influenza A (H1N1) virus infections. These results indicate that LQ has a broad-spectrum inhibitory effect on various viruses by inhibiting viral propagation, impacting immune function, and decreasing inflammatory cytokines.

To our knowledge, few prior published meta-analyses investigated the efficacy of LQ on the rate of conversion to severe cases in their main indication of COVID-19. A meta-analysis by Liu et al. [21] reported that LQ combined with conventional treatment had a lower aggravation rate (RR = 0.59, 95% CI (0.37, 0.94), *P*=0.03). However, the meta-analysis by Liu et al. included 3 RCTs, 3 retrospective case control studies, and 2 retrospective case series with poor quality, which might affect the stability of results. Hu et al. [22] reported that conversion of severe cases (OR = 0.46, 95% CI (0.27, 0.77), *P*=0.0003) were associated with the LQ treatment, but the inclusion of data from 2 RCTs and 2 retrospective case control studies, and enrolled participants including severe cases of COVID-19. Zeng et al. [23] only reported that the disappearance rate of the main clinical symptoms was higher in the LQ group and did not mention the rate of conversion of severe cases. These results indicate a possible association between LQ treatment and decreased rate of conversion of severe cases, but the analyses may have insufficiency of including study quality and statistics to sustain the association.

This meta-analysis, which included 5 RCTs with moderate quality, sought to evaluate the efficacy of LQ on the rate of conversion to severe cases in treating patients with mild or moderate COVID-19. Results of this meta-analysis showed that the combination of LQ and conventional therapy was significantly associated with higher clinical efficacy, rate of CT improvement, and a lower rate of conversion to severe cases. A strength of this meta-analysis is the inclusion of data from RCTs only and included COVID-19 patients without severe cases, which reduced the possibility of confounding variables. Therefore, these results indicated that LQ treatment will have great benefits in reducing the rate of severity and alleviating the deterioration of the patients with mild or moderate COVID-19.

However, the potential mechanism of LQ in reducing the rate of conversion to severe cases in patients with mild or moderate COVID-19 remains uncertain. Recently, network pharmacology by Xia et al. [24] indicates that LQ may target the Akt1 gene by six compounds (beta-carotene, kaempferol, luteolin, naringenin, quercetin, and wogonin) to reduce tissue damage which is induced by COVID-19. At molecular levels, Li et al. [25] have shown that LQ inhibited SARS-CoV-2 replication in Vero E6 cells, resulted in abnormal virion particle morphology in cells, and reduced the mRNA expression of proinflammatory cytokines (TNF-*α*, IL-6, CCL-2/MCP-1, and CXCL-10/IP-10). These results indicate the possible mechanism of LQ in reducing the severity of COVID-19 by inhibiting the inflammatory reaction and regulating the Akt1 pathway.

This study also has several limitations. First, patients with severe COVID-19 were excluded from this study. The efficacy of LQ on all-cause mortality and rate of conversion to mild or moderate cases in severe COVID-19 was unclear. Second, all RCTs were conducted in China, so that the results were regionally restrictive to some extent. Third, some RCTs were not registered and the details of the methods were unclear about performance bias and detection bias.

## 5. Conclusion

In this meta-analysis of RCTs, the combination of LQ and conventional therapy was associated with a lower rate of conversion to severe cases in patients with mild or moderate COVID-19. These results suggest that routine treatment of LQ may be a promising therapy for treating COVID-19.

## Figures and Tables

**Table 1:** Characteristics of included trials.

Included trials	Year	Study design	Country	Male	Age, (y)		LQ	Treatment duration	LQ formulation	LQ dosage
				No. (%)	Control	Intervention	No. (%)	Days		
Chen J. J. [13]	2020	RCT	China	38 (54.3)	45.2 ± 4.7	44.8 ± 4.9	35 (50)	15	Capsules (0.35 g/capsule)	4 capsules, bid
Yu P. [14]	2020	RCT	China	171 (58.0)	47.3 ± 8.7	48.3 ± 9.6	147 (49.8)	7	Granules (6 g/bag)	6 g, tid
Chen C. W. [15]	2021	RCT	China	35 (58.3)	49.5 ± 5.1	50.2 ± 5.1	30 (50)	NA	Capsules (0.35 g/capsule)	4 capsules, tid
Hu K. [16]	2020	RCT	China	150 (52.8)	51.8 ± 14.8	50.4 ± 15.2	142 (50)	14	Capsules (0.35 g/capsule)	4 capsules, tid
Xiao M. Z. [17]	2020	RCT	China	70 (57.9)	53.9 ± 13.9	52.7 ± 14.0	58 (47.9)	14	Granules (6 g/bag)	6 g, tid

LQ: Lianhua Qingwen, RCT: randomized controlled trial, bid: bis in die, tid: ter in die.

## Data Availability

The data supporting the findings of this study will be made available on request.

## Abbreviations

COVID-19: Coronavirus disease 2019
LQ: Lianhua Qingwen capsules or granules
SARS-CoV-2: Severe acute respiratory syndrome coronavirus 2
WOS: Web of Science
CPM: Chinese patent medicines
CNKI: China National Knowledge Infrastructure
CBM: China Biology Medicine
RTCs: Randomized controlled trials
CT: Computer tomography
RR: Relative risk
CI: Confidence interval
WMD: Weighted mean difference.

## Supplementary Materials

Supplementary Materials
*Figure 1*. Ultra-performance liquid chromatography (UPLC) analysis of Lianhuaqingwen. (A) Fingerprint of Lianhuaqingwen (upper panel) and analysis of chemical standards (lower panel). (B) Chemical structures of identified compounds in Lianhuaqingwen, corresponding to the common peak numbers. *Table 1*. Formulation of Lianhuaqingwen capsule (Granule) [1, 2].
