# Supplementary material for: Lianhua Qingwen Capsules Reduced the Rate of Severity in Patients with COVID-19: A System Review and Meta-Analysis of Randomized Controlled Trials
Source: Evid Based Complement Alternat Med. 2022 Feb 2;2022:9617429. doi: 10.1155/2022/9617429 (PMC8812377; doi:10.1155/2022/9617429)
Supplement: Supplementary Materials — Figure 1. Ultra-performance liquid chromatography (UPLC) analysis of Lianhuaqingwen. (A) Fingerprint of Lianhuaqingwen (upper panel) and analysis of chemical standards (lower panel). (B) Chemical structures of identified compounds in Lianhuaqingwen, corresponding to the common peak numbers. Table 1. Formulation of Lianhuaqingwen capsule (Granule) [1, 2]. [file 9617429.f1.zip › 9617429.f1/Supplementary Figure 1 Chemical analysis of Lianhua qingwen.docx]

**A**


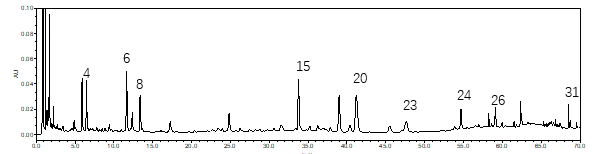


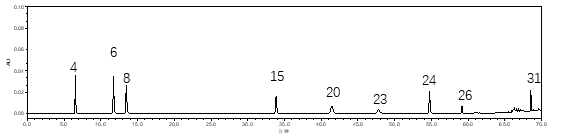


**B**

Fig1. Ultra-performance liquid chromatography (UPLC) analysis of Lianhuaqingwen. (A) Fingerprint of Lianhuaqingwen (upper panel) and analysis of chemical standards (lower panel). (B) Chemical structures of identified compounds in Lianhuaqingwen, corresponding to the common peak numbers.

**Chemical analysis of Lianhuaqingwen**

32 common peaks as well as their retention times and peak area rations on UPLC fingerprint can be used as the important parameters of the quality control for Lianhuaqingwen capsule. 9 of the 32 common peaks were identified by compared with chemical standards, they are Neochlorogenic acid (peak 4), chlorogenic acid (peak 6), cryptochlorogenic acid (peak 8), isoforsythoside A (peak 15), forsythoside A (peak 20), quercitrin (peak 23), isochlorogenic acid C (peak 24), forsythin (peak 26), glycyrrhizic acid (peak 31). Representative chromatograms of standards and tested drug and chemical structures of marker compounds are shown in Fig 1. (Hu et al., 2020)

Hu, K., Guan, W.-j., Bi, Y., Zhang, W., Li, L., Zhang, B., et al. (2020). Efficacy and safety of Lianhuaqingwen capsules, a repurposed Chinese herb, in patients with coronavirus disease 2019: A multicenter, prospective, randomized controlled trial. *Phytomedicine***,** 153242. doi: 10.1016/j.phymed.2020.153242.
