# Supplementary material for: Lianhua Qingwen Capsules Reduced the Rate of Severity in Patients with COVID-19: A System Review and Meta-Analysis of Randomized Controlled Trials
Source: Evid Based Complement Alternat Med. 2022 Feb 2;2022:9617429. doi: 10.1155/2022/9617429 (PMC8812377; doi:10.1155/2022/9617429)
Supplement: Supplementary Materials — Figure 1. Ultra-performance liquid chromatography (UPLC) analysis of Lianhuaqingwen. (A) Fingerprint of Lianhuaqingwen (upper panel) and analysis of chemical standards (lower panel). (B) Chemical structures of identified compounds in Lianhuaqingwen, corresponding to the common peak numbers. Table 1. Formulation of Lianhuaqingwen capsule (Granule) [1, 2]. [file 9617429.f1.zip › 9617429.f1/Supplementary Table 1 Formulation of Lianhua qingwen capsule(Granule).docx]

| Table 1 Formulation of Lianhuaqingwen capsule(Granule)^[1, 2]^ | | |
| --- | --- | --- |
| Ingredient | Components | % |
| *Forsythia suspensa* （Thunb.）Vahl | Dried Fruit | 12.7 |
| *Lonicera japonica* Thunb. | Dried flower bud or opening flower | 12.7 |
| *Ephedra sinica* Stapf | Dried herbaceous stem | 4.2 |
| *Isatis indigotica* Fort. | Dried root | 12.7 |
| *Pogostemon cablin*（Blanco）Benth. | Dried aerial part | 4.2 |
| *Rheum palmatum* L. | Dried root and rhizome | 2.5 |
| *Glycyrrhiza uralensis* Fisch. | Dried root and rhizome | 4.2 |
| *Dryopteris crassirhizoma* Nakai | Dried rhizome and frond bases | 12.7 |
| *Rhodiola crenulata*（Hook. f. et Thoms. ）H. Ohba | Dried root and rhizome | 4.2 |
| *Houttuynia cordata* Thunb. | Dried aerial part | 12.7 |
| *Prunus sibirica* L. | Dried ripe seed | 4.2 |
| Gypsum | CaSO_4_·2H_2_O | 12.7 |
| l-Menthol | C_10_H_20_O | 0.4 |

[1] Hu K, Guan W-j, Bi Y, et al. Efficacy and safety of Lianhuaqingwen capsules, a repurposed Chinese herb, in patients with coronavirus disease 2019: A multicenter, prospective, randomized controlled trial [J]. Phytomedicine, 2020, 153242.

[2] Zhuang W, Fan Z, Chu Y, et al. Chinese Patent Medicines in the Treatment of Coronavirus Disease 2019 (COVID-19) in China [J]. Front Pharmacol, 2020, 11(1066.
